# Combining ability of tropical × temperate maize inducers for haploid induction rate, *R1-nj* seed set, and agronomic traits

**DOI:** 10.3389/fpls.2023.1154905

**Published:** 2023-04-11

**Authors:** Abil Dermail, Thomas Lübberstedt, Willy Bayuardi Suwarno, Sompong Chankaew, Kamol Lertrat, Vinitchan Ruanjaichon, Khundej Suriharn

**Affiliations:** ^1^ Department of Agronomy, Faculty of Agriculture, Khon Kaen University, Khon Kaen, Thailand; ^2^ Department of Agronomy, Iowa State University, Ames, IA, United States; ^3^ Department of Agronomy and Horticulture, Faculty of Agriculture, IPB University, Bogor, Indonesia; ^4^ Plant Breeding Research Center for Sustainable Agriculture, Faculty of Agriculture, Khon Kaen University, Khon Kaen, Thailand; ^5^ National Center for Genetic Engineering and Biotechnology (BIOTEC), 113 Thailand Science Park, Pahonyothin Road, Khlong Nueng, Khlong Luang, Pathum Thani, Thailand

**Keywords:** *Zea mays* L., doubled haploid technology, haploid production, hybrid inducer, heterosis, gene action, heritability, diallel analysis

## Abstract

*In vivo* maternal haploid induction in isolation fields is proposed to bypass the workload and resource constraints existing in haploid induction nurseries. A better understanding of combining ability and gene action conditioning traits related to hybrid inducers is necessary to set the breeding strategy including to what extent parent-based hybrid prediction is feasible. This study aimed to evaluate the following in tropical savanna in the rainy and dry seasons for haploid induction rate (HIR), *R1-nj* seed set, and agronomic traits: 1) combining ability, line per se, and hybrid performance of three genetic pools; 2) genetic parameters, the modes of gene action, and heterosis; and 3) the relationships of inbred–general combining ability (GCA) and inbred–hybrid performance. Fifty-six diallel crosses derived from eight maize genotypes were evaluated in the rainy season of 2021 and the dry season of 2021/2022. Reciprocal cross effects including the maternal effect barely contributed to the genotypic variance for each trait observed. HIR, *R1-nj* seed set, flowering dates, and ear position were highly heritable and additive inherited, while ear length showed dominant inheritance. The equal importance of additive and dominance effects was found for yield-related traits. Temperate inducer BHI306 was the best general combiner for the HIR and *R1-nj* seed set, followed by two tropical inducers, KHI47 and KHI54. The ranges of heterosis were trait-dependent and slightly influenced by the environment, where hybrids in the rainy season consistently had higher heterosis than those in the dry season for each trait observed. Both hybrid groups derived from tropical × tropical and tropical × temperate inducers showed taller plants, larger ear size, and higher seed sets than the corresponding parents. However, their HIRs were still below the standard check of BHI306. The implications of genetic information, combining ability, and inbred–GCA and inbred–hybrid relationships on breeding strategies are discussed.

## Introduction

Maize is the second most important cereal crop in the world due to its multi-purpose as food, feed, and fuel. In Thailand, the use of hybrid seeds has rapidly increased since the late 1990s (Napasintuwong, 2014), and most maize-growing areas are occupied with hybrid cultivars to date. The rising demands on maize hybrid seeds encourage breeders to provide a greater number of inbred lines in a sustainable way. The development of inbred lines as hybrid parents *via* the traditional method is time-consuming, as it requires more than six generations. In contrast, doubled haploid (DH) technology provides fully homozygous DH lines within just two generations (Geiger and Gordillo, 2009). The *in vivo* method for large-scale DH production is widely adopted in maize. It requires four major steps, namely, haploid induction, haploid identification, haploid genome doubling, and fertile haploid (DH_0_) pollination (Chaikam et al., 2019). If the maternal system of haploid induction is applied, haploid inducers should be assigned as male to induce haploids from the donor germplasm as female. Haploid induction is commonly performed by hand pollination, making this method laborious and resource-intensive. Wind pollination in isolation fields could be considered by implementing methods used for maize hybrid seed production (MacRobert et al., 2014) by assigning a couple of inducer rows as pollinators and several rows of donor germplasm as seed parents.

Haploid induction rate (HIR) is one of the major traits related to haploid inducers. To date, three genes responsible for HIR have been isolated including *mtl* (Gilles et al., 2017; Kelliher et al., 2017; Liu et al., 2017), *zmdmp* (Zhong et al., 2019), and *zmpld3* (Li et al., 2021). In addition, since numerous haploid induction crosses are required to determine HIR among large numbers of inducers, the method for haploid identification should be practical, resource-saving, and doable at the earliest stage. The *R1-nj* anthocyanin marker is widely used in ploidy discrimination in maize (Chaikam et al., 2019), allowing operators to visually identify haploids and diploids at the kernel stage. Diploids will express a purple pigmentation on the scutellum embryo and aleurone layer, while haploids will show a colorless embryo and purple pigmentation on the aleurone endosperm (Nanda and Chase, 1966). Thus, haploid inducers carrying the *R1-nj* marker facilitate haploid selection without the need for sophisticated equipment.

The tropical savanna (TS) climate of Thailand has seasonal variation, spanning from summer dry (February–May) and rainy (May–October) to cool dry (October–February), but only the last two are good for maize production. The Plant Breeding Research Center for Sustainable Agriculture of Khon Kaen University has initiated breeding haploid inducers using public inducer genotype “Stock 6”, and currently, the first haploid inducers for the regions under TS have been developed, but the HIRs are still low (Dermail et al., 2021). Modern haploid inducers have higher HIRs ranging from 8% to 14% (Liu et al., 2016). Temperate inbred inducer BHI306 derivatives reached HIR > 20% in the summer nursery of 2022 (U.K. Frei 2022, personal communication; Y.R. Chen 2022, personal communication). Genotype BHI306, however, lacks tropical adaptability to the rainy season and performs slightly better in the cool dry season when first introduced in Khon Kaen, Thailand. The maladaptation syndrome including short stature, poor pollen production and seed set, and susceptibility to several tropical diseases is often recognized when temperate inducers are grown in the tropics (Prasanna, 2012; Prigge et al., 2012a). Thus, tropicalization for regional adaptation is a major concern after HIR in breeding haploid inducers. Introgression of HIR from BHI306 into our semi-tropically adapted inducers and other tropical non-inducer maize germplasm is expected to achieve those goals.

Most available haploid inducers are inbreds (Liu et al., 2016), while donor germplasm is typically either F_1_ or F_2_ materials (Couto et al., 2019), which may result in inefficient pollination. Vigorous inducer genotypes with high HIR can enhance the efficiency of DH procedures not because of higher success rates of pollination and seed set only but because of bypassing the workload and resources required in induction nurseries. Heterosis, a better F_1_ performance than their parents (Hallauer et al., 2010), is exploited in maize hybrid breeding (Fu et al., 2014). Positive heterosis has previously been reported for maize characteristics related to agronomic adaptation such as plant height, ear position, yield components, and grain yield (Flint-Garcia et al., 2009; Wei et al., 2016; Yu et al., 2020), although the magnitudes within traits vary depending on genetic background and environment (Moll et al., 1965; Li et al., 2018). Perhaps hybrid inducers can also benefit from hybrid vigor. However, there is a lack of information regarding heterosis for HIR and *R1-nj* expression in maize. The systematic introgression of exotic lines into local germplasm in target environments requires adequate information on breeding values and genetic parameters of the selected genotypes through combining ability studies (Nelson and Goodman, 2008). Sprague and Tatum (1942) proposed general combining ability (GCA) and specific combining ability (SCA) for selecting promising parental lines and hybrids, respectively. Since hybrid formations followed by extended field trials are resource-intensive, the important questions are as follows: 1) should reciprocal crosses be included in hybrid formation?; 2) is line per se evaluation reliable to predict GCA and hybrid performance?; 3) what is the mode of inheritance of traits important for haploid inducers? Diallel analysis method 1 (Griffing, 1956) allows breeders to make all possible crosses among certain genotypes and to investigate the reciprocal cross effects including the maternal effect (Cockerham, 1963). This study aimed to evaluate the following in tropical savanna in the rainy and dry seasons for HIR, *R1-nj* seed set, and agronomic traits: 1) combining ability, line per se, and hybrid performance of three genetic pools (temperate inducer, tropical inducer, and tropical non-inducer); 2) genetic parameters, the modes of gene action, and heterosis; and 3) the relationships of inbred–GCA and inbred–hybrid performance. Information obtained in this study can be applied in breeding strategies involving traits related to haploid induction ability and tropical adaptation and to what extent the hybrid prediction based on inbred performance on given traits reduces the need for hybrid formation and evaluation.

## Materials and methods

### Plant materials and hybrid formation

Eight maize genotypes were used for F_1_ hybrid formation (**Table 1**). Those genotypes comprised five tropical inbred inducers (KHI42, KHI47, KHI49, KHI54, and KHI59), one temperate inbred inducer (BHI306), and two tropical non-inducers (hybrid S7328 and inbred Takfa1). Those five tropical inbred inducers had inducer Stock 6 as the founder parent for haploid induction ability and were developed by the Plant Breeding Research Center for Sustainable Agriculture of Khon Kaen University in Thailand (Dermail et al., 2021). Inbred inducer, BHI306, had inducers RWS and RWK-76 as the founder parents for haploid induction ability and was developed by the DH Facility of Iowa State University (DHF-ISU) (https://www.doubledhaploid.biotech.iastate.edu/). Maize inbred Takfa1 was a public source germplasm and developed by the Nakhon Sawan Research Center, Thailand, whereas F_1_ hybrid S7328 is drought resistant and was developed by Syngenta Seeds, Thailand. Since those non-inducer parents had good agronomic performance but do not carry the *R1-nj* purple embryo marker and do not have haploid induction ability, they were used as positive checks for agronomic traits and negative checks for HIR and the *R1-nj* expression. Two commercial F_1_ hybrid cultivars, S7328 and P789, were assigned as donor females for haploid induction. Genotype P789 was developed by Pacific Seeds, Thailand. The reason for using those genotypes was that they produced mostly large and flat embryos, facilitating visual scoring for the *R1-nj* kernel marker.

**Table 1:** Brief descriptions of six haploid inducers used in this study.

Genotype	Type	Haploid induction ability	Purple embryo marker	Agronomic performance^1^	Ear size
KHI42	Inbred	Yes (*mtl*, *ZmDMP*)	*R1-nj*	Good	Medium
KHI47	Inbred	Yes (*mtl*, *ZmDMP*)	*R1-nj*	Good	Medium
KHI49	Inbred	Yes (*mtl*, *ZmDMP*)	*R1-nj*	Good	Medium
KHI54	Inbred	Yes (*mtl*, *ZmDMP*)	*R1-nj*	Good	Small
KHI59	Inbred	Yes (*mtl*, *ZmDMP*)	*R1-nj*	Good	Big
BHI306	Inbred	Yes (*mtl*, *zmdmp*)	*R1-nj*	Poor	Small
Takfa1	Inbred	No (MTL, ZMDMP)	*r1-nj*	Excellent	Small
S7328	Hybrid	No (MTL, ZMDMP)	*r1-nj*	Excellent	Large

^1^Evaluated under field conditions in the typical growing seasons of tropical Thailand.

The parents were crossed in an 8 × 8 full diallel mating design to generate 56 F_1_ progenies including the reciprocals, which were composed of 10, 20, 20, 4, and 2 crosses of tropical inducer × temperate inducer, tropical inducer × tropical inducer, non-inducer × tropical inducer, non-inducer × temperate inducer, and non-inducer × non-inducer, respectively. Those hybrid formations were performed in the late dry season of 2020/2021 at the Agronomy Field Crop Station of Khon Kaen University in Khon Kaen, Thailand.

### Experimental design and haploid induction

The experiment was conducted in the rainy season of 2021 and the dry season of 2021/2022 at the Agronomy Field Crop Station of Khon Kaen University (16°28′27.7″N, 102°48′36.5″E; 190 m above sea level) in Khon Kaen, Thailand. In both growing seasons, an 8 × 8 lattice design with three replications was used. To minimize the effects of root and shade competitions due to the better vigor of F_1_ progenies, parents were separately randomized and placed in the first block within replication and season. The plot size was two rows of 4-m length with 75 cm × 25 cm plant spacing producing 30–32 plants per plot. The crop field management followed the Department of Agriculture, Thailand, recommendations (2021) including fertilization, irrigation, and control of pests, diseases, and weeds.

To evaluate the haploid induction rate, about five to six induction crosses per donor per replication per season were performed. The donor plants were placed adjacent to the inducer plots and were planted for four staggered planting dates with a 5-day interval to ensure nicking. To avoid pollen contamination, routine plant checking in donor plants was performed by shoot bagging and detasseling.

### Data collection

The seeds of each donor’s ear were manually sorted *via* the *R1-nj* marker. This system distinguished the ploidy levels at the seed stage based on anthocyanin pigmentation on the crown endosperm and scutellum embryo (Nanda and Chase, 1966). Putative diploid seeds displayed a purple crown and scutellum, whereas putative haploid seeds showed a purple crown and colorless embryo.

HIR was calculated as the percentage of putative haploid seeds per induction cross, as follows:


$$\mathrm{HIR (\%) =}\frac{\mathrm{seed number of putative haploid}}{\text{seed set}}\text{x }100$$

where the seed set was calculated by adding the number of putative haploid seeds, putative diploid seeds, and the seeds without the *R1-nj* expression.

The *R1-nj* seed set of inducers (ISR) and donors (DSR) were observed to quantify the expression of the *R1-nj* marker in the inducer and induced donor ears, respectively, by dividing the number of putative diploid and haploid seeds by the total seed number per ear. The sample size per plot was 20 inducer ears and five to six induced donor ears for ISR and DSR, respectively.

Ten inducer plants per plot were used for measuring plant height (cm), from ground level to the base of the tassel after the milk stage and ear height (cm), from ground level to the node bearing the uppermost ear after the milk stage. Anthesis date (the number of days from sowing to when 50% of the plants have shed the pollen) and silking date (the number of days from sowing to when silks have emerged on 50% of the plants) were recorded on a plot basis. Ten inducer ears were measured for yield components after harvest at physiological maturity (R_6_ stage), including ear length (cm), ear diameter (cm), ear weight (g), total kernel weight per ear (g), and total kernel number per ear.

### Statistical analysis

The diallel analysis regarding Griffing (1956) method 1 and mixed B model was chosen by considering genotype as a fixed effect, while season, replication within season, and block within replication and season were the random effects. The sums of squares for hybrids and hybrids × seasons were partitioned into GCA and SCA, and their interaction with the season (GCA × season and SCA × season), respectively. The linear model for combined analysis of variance multi-season in lattice design (Rodríguez et al., 2018) was as follows:


$$y_{ijkd}=\;µ\;+\;e_{d}+\;REP_{k}\left(e_{d}\right)\;+\;BLK\left(REP_{k}e_{d}\right)\;+\;gca_{i}+\;sca_{ij}+\;m_{i}+\;r_{ij}+\;e_{d}gca_{i}+\;e_{d}sca_{ij}+\;e_{d}m_{i}+\;e_{d}r_{ij}+\;\varepsilon_{ijkl}$$

where *y_ijk_* is the observed value, *µ* is the general mean, *e_d_* is the effect of season *d*, *REP_k_* (*e_d_*) is the effect of replicate *k* nested in season *d*, *gca_i_* is the general combining ability effect, *sca_ij_* is the specific combining ability effect, *m_i_* is the maternal effect, *rij* is the reciprocal effect, *e_d_gca_i_* is the effect of interaction between season *d* and GCA, *e_d_sca_i_* is the effect of interaction between season *d* and SCA, *e_d_m_i_* is the effect of interaction between season *d* and maternal, *e_d_r_ij_* is the effect of interaction between season *d* and reciprocal, *BLK*(*REP_k_ e_d_*) is the random effect of block nested in replicate *k* nested in environment *d*, and *ϵ_ijkl_* is the residual.

Combining ability estimates (GCA and SCA) including their standard errors were calculated following Singh and Chaudhary (1979). Variance components through model II diallel analysis by assuming all factors as random effects were estimated to determine gene action and narrow-sense heritability. Baker’s ratio was calculated based on the relative importance of GCA/SCA variances (Baker, 1978). The estimates of broad- and narrow-sense heritability were calculated on a plot basis (Holland et al., 2003). Mid-parent heterosis (MPH) and best-parent heterosis (BPH) were calculated using the means of parents and hybrids following Rai (1979). The above analyses were performed by using the software AGD-R 5.0 (Rodríguez et al., 2018). Means were compared using Tukey’s honestly significant difference (HSD) test at a 5% probability level.

## Results

### Diallel analysis

Season effect was significant for all traits except for the *R1-nj* seed set with donor P789 (DSR_P) (**Table 2**). Genotype was significant for all traits. Both GCA and SCA were significant for all traits, while reciprocal was significant for all traits except for ear diameter (ED). Maternal was significant for all traits except for ED, while non-maternal was significant for all traits except for ear length (EL) and ED. The interaction between genotype and season (G × S) was significant for all traits except for DSR_P. While GCA × S was significant for all traits except for DSR_P, SCA × S was significant for all traits except for silking date (SD), EL, and DSR_P. Reciprocal × S, maternal × S, and non-maternal × S were significant for several traits observed.

**Table 2:** Diallel analysis for agronomic traits, *R1-nj* seed set, and haploid induction rate of 8 parental genotypes and 56 F_1_ progenies evaluated in the rainy season of 2021 and the dry season of 2021/2022.

Source of variation	df	PH	EH	AD	SD	EL	ED	EW
Season (S)	1	128,331.1**	69,475.2**	8,578**	10,689**	148.9**	15.6**	158,898.1**
Replication (R)/S	4	360.7**	232.3**	6**	5*	2.7 ns	1.4 ns	496.1*
Block/R S	42	247.5**	178.9**	3**	3**	1.9 ns	1.5 ns	294.7**
Genotype	63	2,996.6**	2,008.9**	87**	91**	14.2**	1.7*	5,062.6**
GCA	7	14,121.2**	11,228.5**	608**	597**	39.7**	3.9**	20,559.5**
SCA	28	2,581.7**	1,171.5**	38**	50**	19.1**	2.8**	5,609.2**
Reciprocal	28	631.1**	546.9**	6**	7**	2.9*	0.1 ns	646.2**
Maternal	7	1,050.1**	1,171.9**	5**	4**	5.2**	0.2 ns	1,068.9**
Non-maternal	21	491.5**	338.6**	6**	8**	2.1 ns	0.1 ns	505.3**
Genotype × S	63	125.1**	106.2**	3**	6**	3.6**	1.6*	346.2**
GCA × S	7	155.0*	136.9**	9**	33**	12.3**	3.0*	486.3**
SCA × S	28	175.9**	141.2**	3**	2 ns	2.3 ns	2.8**	360.2**
Reciprocal × S	28	64.2 ns	57.2 ns	2 ns	3**	2.8*	0.1 ns	283.8*
Maternal × S	7	155.3*	155.1**	1 ns	4*	1.5 ns	0.1 ns	124.9 ns
Non-maternal × S	21	33.8 ns	24.5 ns	2 ns	3*	3.2*	0.1 ns	336.7**
cv, %		4.7	7.3	2.0	2.0	8.8	29.3	11.0
Source of variation	df	KW	TK	ISR	DSR_P	DSR_S	HIR_P	HIR_S
Season (S)	1	127,409.2**	79,686.5**	6,421.6**	19.6 ns	20,171.8**	2.3**	2.6**
Replication (R)/S	4	178.3 ns	585 ns	6.1 ns	413.9**	176.5 ns	0.1 ns	0.1 ns
Block/R S	42	180.0*	698 ns	22.3 ns	117.8**	92.7 ns	0.1 ns	0.2*
Genotype	63	3,487.8**	35,761**	4,547.9**	6,688.3**	4,078.9**	8.8**	5.4**
GCA	7	13,879.9**	162,604**	37,285.4**	47,969.0**	29,671.6**	60.1**	32.4**
SCA	28	3,944.8**	36,495**	705.7**	2,258.1**	1,272.3**	3.7**	3.1**
Reciprocal	28	435.9**	3,315**	205.2**	786.1**	450.9**	1.0**	0.9**
Maternal	7	806.7**	6,416**	374.9**	1,488.5**	503.9**	0.8**	0.7**
Non-maternal	21	312.3**	2,282**	148.6**	552.0**	433.3**	1.1**	1.0**
Genotype × S	63	231.6**	2,612**	218.1**	3.3 ns	446.6**	0.3**	0.3**
GCA × S	7	379.1**	3,084**	766.7**	0.1 ns	1,356.3**	0.3**	0.4**
SCA × S	28	238.7**	3,228**	187.2**	0.1 ns	392.2**	0.5**	0.3**
Reciprocal × S	28	182.1*	1,855**	100.7**	12.2 ns	259.3**	0.2**	0.2 ns
Maternal × S	7	94.1 ns	2,262**	40.8 ns	22.6 ns	298.9*	0.2*	0.1 ns
Non-maternal × S	21	211.4**	1,720**	120.6**	8.8 ns	246.1*	0.2**	0.2*
cv, %		11.2	8.0	7.1	14.7	26.8	24.8	28.7

PH, plant height; EH, ear height; AD, anthesis date; SD, silking date; EL, ear length; ED, ear diameter; EW, ear weight; KW, total kernel weight per ear; TK, total kernel number per ear; ISR, the *R1-nj* seed set of inducers; DSR_P, the *R1-nj* seed set with donor P789; DSR_S, the *R1-nj* seed set with donor S7328; HIR_P, haploid induction rate with donor P789; HIR_S, haploid induction rate with donor S7328; ns, not significant.

**significant at *p* ≤ 0.01; *significant at *p* ≤ 0.05.

### Variance components, heritability, and gene action

The additive variance was higher than the dominance variance for EH, AD, SD, ISR, DSR_P, DSR_S, HIR_P, and HIR_S (**Table 3**). Those traits, except for ED, PH, and EH, had high Baker’s ratios and estimates of broad- and narrow-sense heritability above 0.70, 0.80, and 0.60, respectively. The dominance variance was higher than the additive variance for EL, EW, KW, and TK. Those traits had low Baker’s ratios and narrow-sense heritability below 0.50. The other two traits, PH and ED, had nearly equal estimates between additive and dominance variances. The maternal variance was zero for AD, SD, ED, HIR_P, and HIR_S. The non-maternal variance was slightly higher than the maternal variance for all traits except for EL and ISR.

**Table 3:** Variance components and heritability estimates for agronomic traits, *R1-nj* seed set, and haploid induction rate of 8 parental genotypes and 56 F_1_ progenies evaluated in the rainy season of 2021 and the dry season of 2021/2022.

Trait	Importance of additive/dominance			Reciprocal		Heritability			
	$\sigma_{A}^{2}$	$\sigma_{D}^{2}$	Baker’s ratio	$\sigma_{m}^{2}$	$\sigma_{nm}^{2}$	$\sigma_{G}^{2}$	$\sigma_{P}^{2}$	$h_{b}^{2}$	$h_{n}^{2}$
Plant height	241.72	225.11	0.52	4.55	38.14	466.83	587.78	0.79	0.41
Ear height	209.99	96.40	0.69	7.32	26.17	306.38	391.97	0.78	0.54
Anthesis date	11.75	3.32	0.78	0.00	0.36	15.07	16.69	0.90	0.70
Silking date	10.77	4.45	0.71	0.00	0.43	15.23	17.16	0.89	0.63
Ear length	0.23	1.57	0.13	0.05	0.00	1.80	3.54	0.51	0.06
Ear diameter	0.02	0.00	0.96	0.00	0.00	0.02	1.46	0.01	0.01
Ear weight	310.75	491.14	0.39	8.08	14.04	801.90	998.70	0.80	0.31
Total kernel weight per ear	205.41	346.77	0.37	6.37	8.41	552.18	680.20	0.81	0.30
Total kernel number per ear	2,642.43	3,112.70	0.46	37.41	46.86	5,755.13	6,501.24	0.89	0.41
The *R1-nj* seed set of inducers	750.19	48.52	0.94	3.19	2.33	798.71	831.38	0.96	0.90
The *R1-nj* seed set with donor P789	954.40	211.34	0.82	9.61	45.27	1,165.74	1,279.25	0.91	0.75
The *R1-nj* seed set with donor S7328	571.89	82.35	0.87	0.19	15.60	654.24	801.67	0.82	0.71
HIR with donor P789	1.18	0.30	0.80	0.00	0.07	1.48	1.63	0.91	0.72
HIR with donor S7328	0.61	0.26	0.70	0.00	0.07	0.87	1.05	0.83	0.58

$\sigma_{A}^{2},\;\sigma_{D}^{2},\;\sigma_{m}^{2},\;\sigma_{nm}^{2},\;\sigma_{G}^{2}$ and $\sigma_{P}^{2}$ indicate variance of additive, dominance, maternal, no maternal, genotypic, and phenotypic, respectively; $h_{b}^{2}$ and $h_{n}^{2}$ indicate broad-sense and narrow-sense heritabilities, respectively; HIR indicates haploid induction rate. Baker’s ratio = 2σGCA/(2σGCA + σSCA), where σGCA and σSCA are variance of general combining ability and specific combining ability, respectively.

Considering genetic parameters, the relative importance of GCA/SCA through the Baker’s ratio, and heritability estimates, our study indicated the following: 1) additive gene action played a significant role in ear position, flowering dates, the *R1-nj* expression in both inducer and donor kernels, and HIR; 2) dominance gene action was more important for ear length; 3) both additive and dominance were equally important to control plant height, ear weight, total kernel weight per ear, and total kernel number per ear; and 4) reciprocal variance, composed of maternal and non-maternal variances, barely contributed to the total genotypic variance on each trait observed.

### General combining ability and per se performance

The estimates of GCA were highly variable within and across traits observed (**Table 4**). Non-inducer genotypes, namely, S7328 and Takfa1, had significant and positive GCA for PH, EH, AD, SD, EL, ED, EW, KW, and TK. In this regard, genotype S7328 was a better combiner than Takfa1 for agronomic traits. Inducer genotypes including BHI306 and five KHI families had significant and positive GCA for ISR, DSR_P, DSR_S, HIR_P, and HIR_S, while non-inducer genotypes had significant and negative GCA for those traits. Among inducer genotypes, BHI306 and KHI47 were the best combiners for HIR-related traits. Since high line per se and positive GCA are in a favorable direction for parental selection, different gene pools contributed genetic potential and favorable alleles for different traits, as follows: temperate inbred inducer for improving HIR and *R1-nj* anthocyanin marker expression; tropical inbred inducers for improving agronomic adaptation, HIR, and the *R1-nj* expression; and tropical non-inducers for improving agronomic adaptation only.

**Table 4:** Estimates of GCA effects and per se performance for agronomic traits, *R1-nj* seed set, and haploid induction rate of eight parents evaluated in the rainy season of 2021 and the dry season of 2021/2022.

Parent	Height (cm)				Days after planting (DAP)				Ear					
	Plant		Ear		Anthesis		Silking		Length (cm)		Diameter (cm)		Weight (g)	
	Per se	GCA	Per se	GCA	Per se	GCA	Per se	GCA	Per se	GCA	Per se	GCA	Per se	GCA
BHI306	94.0	−19.65**	27.8	−22.55**	47.7	−5.80**	46.2	−5.89**	9.0	−1.08 **	2.9	−0.29 **	28.3	−20.65 **
KHI42	134.0	−5.84**	64.3	0.01 ns	55.0	−0.35**	54.8	−0.42**	11.7	−0.04 ns	3.1	−0.03 ns	44.8	1.34 *
KHI47	186.0	14.59**	86.8	8.17**	59.2	2.04**	60.8	2.18**	13.6	0.35 **	3.5	−0.14 *	73.1	−4.70 **
KHI49	153.1	−0.98*	70.6	2.47**	58.8	0.50**	62.0	1.04**	11.8	−0.01 ns	3.7	0.33 **	65.5	−4.20 **
KHI54	120.2	−10.10**	56.2	−7.03**	58.3	−0.20*	59.8	0.30**	9.7	−0.47 **	3.6	−0.04 ns	58.1	−4.65 **
KHI59	154.3	3.67**	72.5	3.51**	57.8	0.37**	59.5	0.71**	12.6	0.39 **	3.4	−0.08 ns	60.8	0.96 ns
S7328	202.0	16.66**	110.7	13.04**	59.7	1.78**	58.3	1.20**	16.1	1.08 **	4.7	0.26 **	176.6	31.76 **
Takfa1	151.5	1.66**	78.8	2.37**	60.5	1.66**	57.7	0.87**	11.3	−0.23 *	3.7	−0.01 ns	64.9	0.13 ns
Mean	149.4		71.0		57.1		57.4		12.0		4.0		71.5	
HSD 5%	31.0		15.2		2.8		3.3		3.5		0.3		23.8	
SE		0.82		0.64		0.11		0.12		0.12		0.11		1.24
r	0.99**		0.97**		0.97**		0.95**		0.97**		0.74*		0.93**	
Parent	Total kernel per ear				The *R1-nj* seed set (%)						Haploid induction rate (%)			
	Weight (g)		Number		Inducer		Donor P789		Donor S7328		Donor P789		Donor S7328	
	Per se	GCA	Per se	GCA	Per se	GCA	Per se	GCA	Per se	GCA	Per se	GCA	Per se	GCA
BHI306	13.1	−16.59**	83.5	−68.89**	100.0	18.44**	73.2	15.33**	35.9	11.58 **	3.5	1.17 **	1.5	11.58 **
KHI42	34.1	0.00 ns	121.6	−4.00*	91.9	2.78**	96.6	14.09**	62.0	9.53 **	1.1	0.08 **	0.5	9.53 **
KHI47	54.7	−5.35**	206.1	−22.27**	98.6	13.01**	96.0	15.62**	79.2	11.80 **	2.0	0.41 **	1.3	11.80 **
KHI49	44.6	−4.43**	187.9	11.66**	74.2	12.52**	36.4	11.03**	49.7	12.28 **	1.4	0.12 **	0.7	12.28 **
KHI54	43.5	−4.24**	171.2	−27.71**	85.8	7.01**	95.1	4.96**	63.4	3.98 **	2.4	0.42 **	1.7	3.98 **
KHI59	48.6	3.81**	205.1	21.92**	73.3	8.23**	86.8	10.49**	71.7	7.09 **	0.7	0.09 **	0.5	7.09 **
S7328	137.4	25.59**	450.9	69.42**	0.0	−32.83**	0.0	−37.53**	0.0	−28.03 **	0.0	−1.15 **	0.0	−28.03 **
Takfa1	51.5	1.21*	243.1	19.87**	0.0	−29.15**	0.0	−33.99**	0.0	−28.23 **	0.0	−1.15 **	0.0	−28.23 **
Mean	53.4		208.7		65.5		60.5		45.2		1.4		0.8	
HSD 5%	25.9		81.6		7.5		17.6		29.7		0.9		0.8	
SE		0.99		2.39		0.47		0.75		1.10		0.03		1.10
r	0.94**		0.86**		0.96**		0.87**		0.87**		0.93**		0.69 ns	

HSD 5% was used as critical value for per se; SE was assigned as critical value for GCA estimates; ** and * indicate GCA estimates significantly different from zero at ≥2SE and ≥SE, respectively; ns GCA indicates estimates not significantly different from zero at ≥SE; ** and * r indicates significance at *p* ≤ 0.01 and *p* ≤ 0.05, respectively.

GCA, general combining ability; r, Pearson’s linear correlation coefficient; HSD, Tukey’s honestly significant difference test; SE, standard error; ns, not significant.

Correlation coefficients between GCA estimates and per se for all traits were significant and high above 0.86, except for HIR_S and ED. This suggested that predicting good general combiners for hybrid formation based on line per se was reliable for all maize traits studied, except for ear diameter and HIR with donor genotype S7328.

### Heterosis and hybrid performance

**Figure 1:**
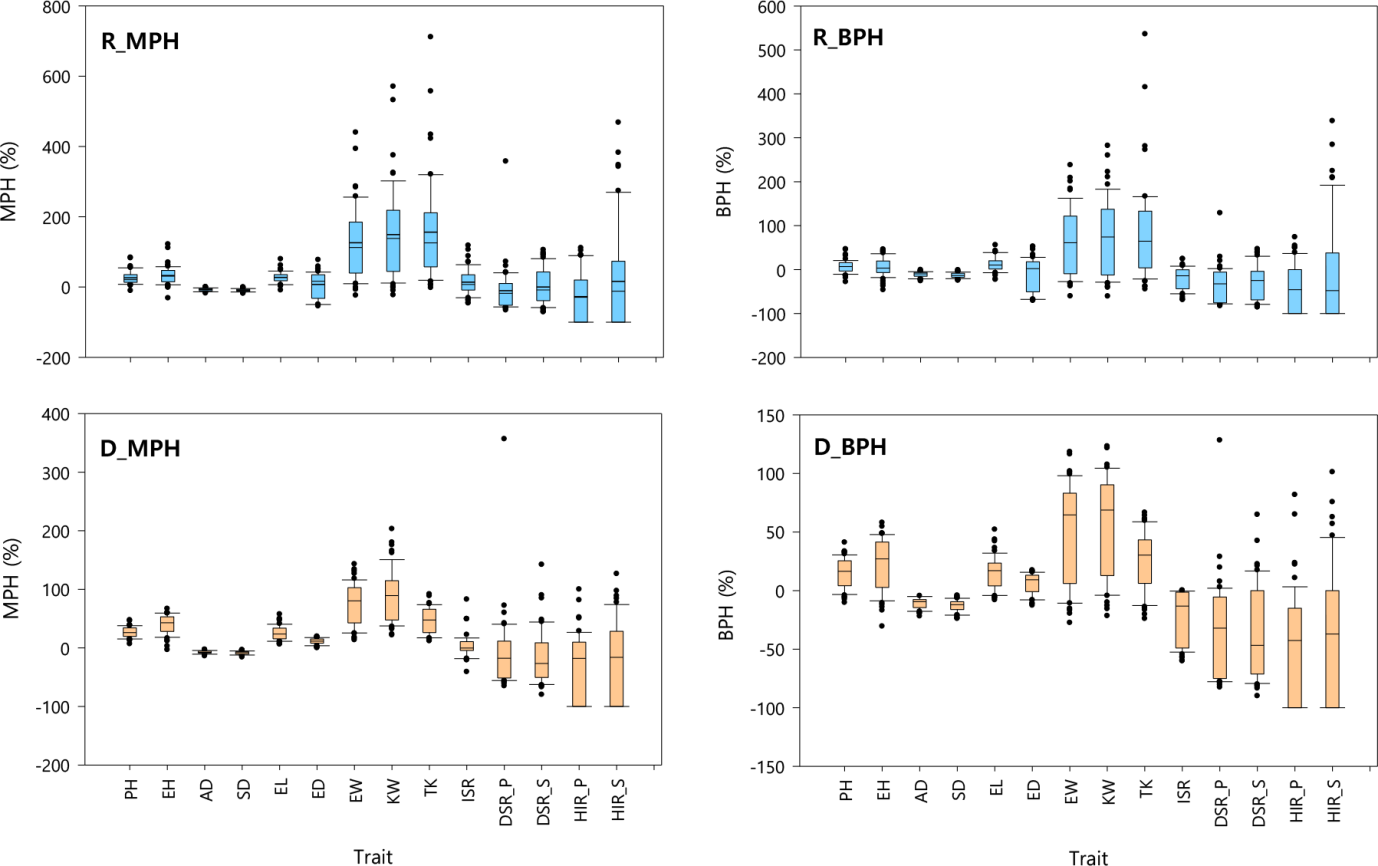
Heterosis performance of 56 crosses in the rainy (R_MPH and R_BPH) and dry (D_MPH and D_BPH) seasons. MPH, mid-parent heterosis; BPH, best-parent heterosis; PH, plant height; EH, ear height; AD, anthesis date; SD, silking date; EL, ear length; ED, ear diameter; EW, ear weight; KW, total kernel weight per ear; TK, total kernel number per ear; ISR, the *R1-nj* seed set of inducers; DSR_P, the *R1-nj* seed set with donor P789; DSR_S, the *R1-nj* seed set with donor S7328; HIR_P, haploid induction rate with donor P789; HIR_S, haploid induction rate with donor S7328.

In the rainy season, the means and the ranges of both MPH and BPH were high for TK, KW, and EW; moderate for EH, EL, PH, HIR_S, and ISR; and low for ED, DSR_S, AD, SD, DSR_P, and HIR_P (**Figure 1**). In the dry season, the magnitudes of both MPH and BPH were high for KW and EW; moderate for TK, EH, PH, EL, and ED; and low for ISR, AD, SD, DSR_P, DSR_S, HIR_S, and HIR_P. The results indicated that the magnitudes of heterosis in maize largely depended upon traits and may be affected by different growing environments. For instance, the rainy season led to higher heterosis than the dry season.

**Figure 2:**
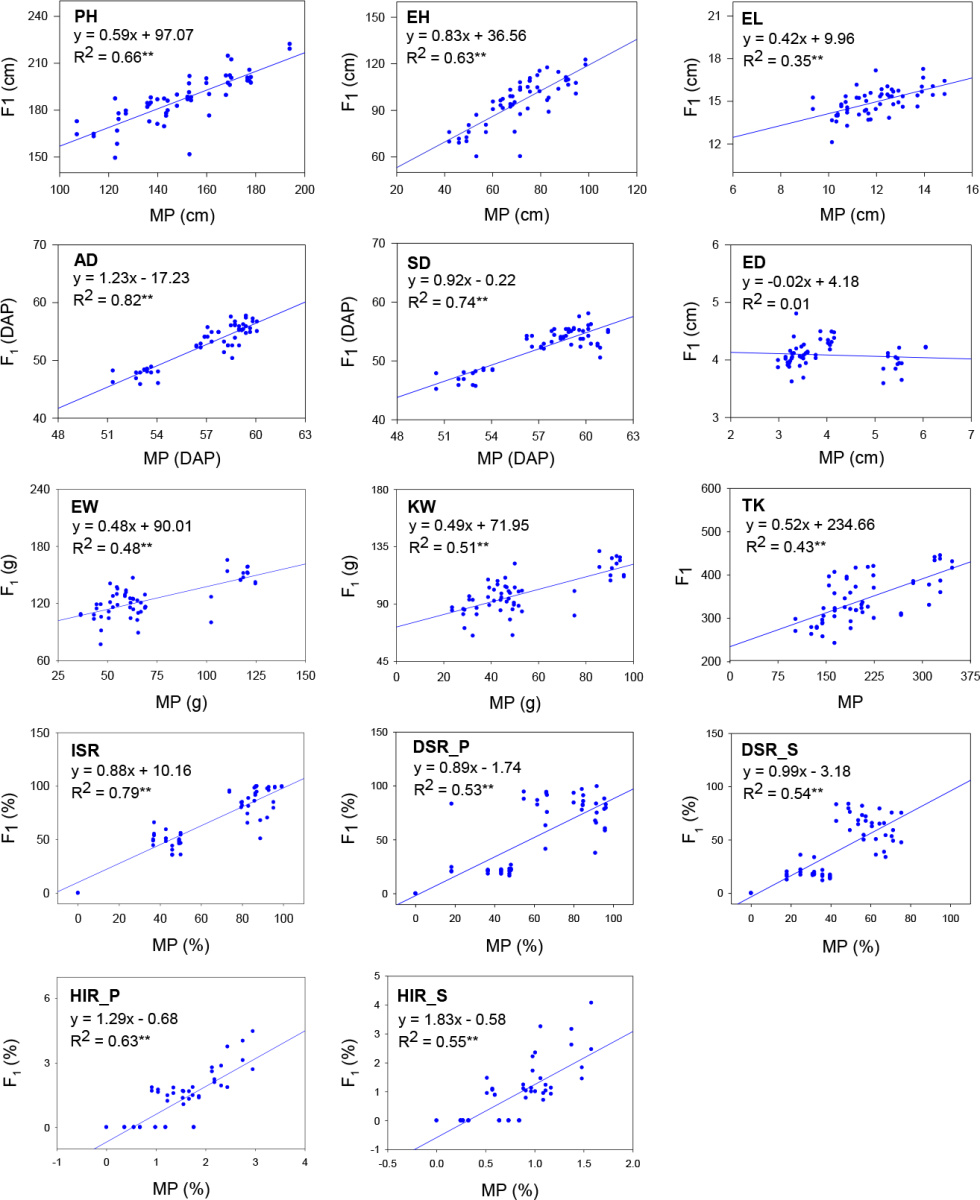
Linear regression between mid-parent (MP) and hybrid performance (F_1_) of 56 crosses evaluated across two seasons. PH, plant height; EH, ear height; AD, anthesis date; SD, silking date; EL, ear length; ED, ear diameter; EW, ear weight; KW, total kernel weight per ear; TK, total kernel number per ear; ISR, the *R1-nj* seed set of inducers; DSR_P, the *R1-nj* seed set with donor P789; DSR_S, the *R1-nj* seed set with donor S7328; HIR_P, haploid induction rate with donor P789; HIR_S, haploid induction rate with donor S7328. R^2^ coefficient of determination. ** significant at *p* ≤ 0.01.

**Figure 3:**
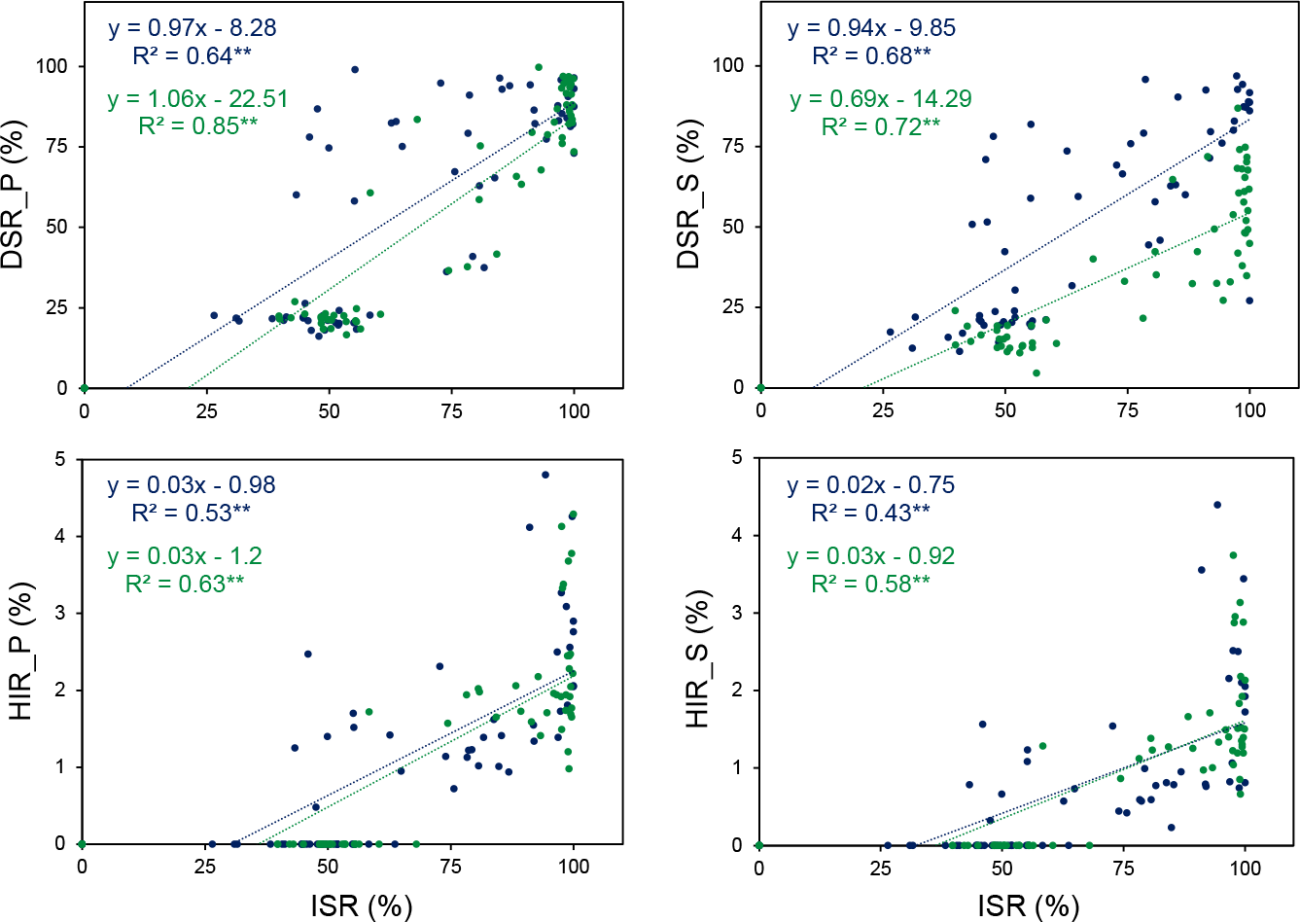
Linear regression between the *R1-nj* seed set of inducers (ISR) and the *R1-nj* seed set with two donors (P789 (DSR_P) and S7328 (DSR_S)) and haploid induction rate with two donors (P789 (HIR_P) and S7328 (HIR_S)) of 64 genotypes in the rainy (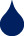) and dry (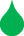) seasons. ** significant at *p ≤ 0.01*.

The coefficient (b) and the R-square (R^2^) values for linear regression between mid-parent and hybrid performance were high for AD, SD, and ISR; moderate for PH, EH, EW, KW, TK, DSR_P, DSR_S, HIR_P, and HIR_S; and low for EL (**Figure 2**). This illustrates that the mid-parent value could be a good predictor to estimate the hybrid performance for flowering time and the *R1-nj* expression, but the predictability seems not sufficient for yield components and HIR. In addition, the *R1-nj* seed set of inducers (ISR) had good predictability for DSR_P and DSR_S but not for HIR_P and HIR_S (**Figure 3**). This indicates that inducer genotypes with intense *R1-nj* expression would facilitate haploid selection based on the *R1-nj* kernel marker. However, it does not equal high HIR.

**Figure 4:**
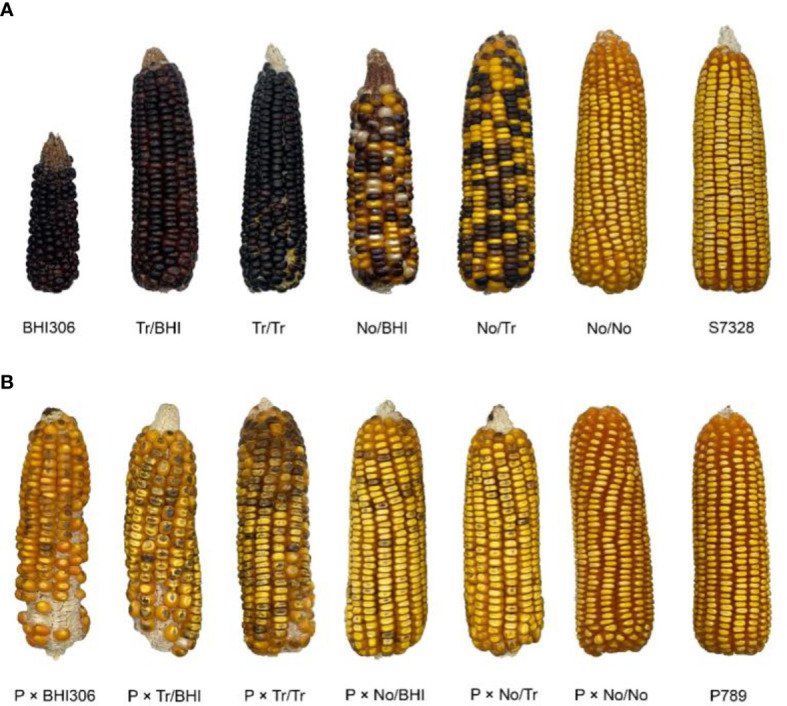
The expression of kernel *R1-nj* anthocyanin marker in pollinator ears **(A)** and donor genotype P789 pollinated with respective pollinators **(B)**. List of pollinator genotypes from left to right: inbred inducer BHI306, KHI54 × BHI306 (representative of Tr/BHI group), KHI47 × KHI49 (representative of Tr/Tr group), Takfa1 × BHI306 (representative of No/BHI group), S7328 × KHI47 (representative of No/Tr group), and Takfa1 × S7328 (representative of No/No group). Genotypes S7328 and P789 were used as negative checks for *R1-nj* anthocyanin marker in pollinators **(A)** and induced donor ears **(B)**, respectively.

The average Tr/BHI hybrid mean was significantly higher than Tr/Tr hybrid means and per se of tropical inbred inducers but lower than per se of BHI306 for HIR (**Table 5**). Any crosses involving non-inducer genotypes resulted in null HIR like Takfa1 and S7328 genotypes as negative checks for HIR. This indicates that hybrid inducers require an equal presence of alleles responsible for HIR and that including BHI306 in such a crossing scheme could further enhance the haploid frequency. Both ISR and DSR of Tr/BHI hybrids were the highest among other hybrid groups but comparable to per se of inducer parents either BHI306 or tropical inbred inducers. The hybrid means between the No/BHI and No/Tr groups were slightly different for ISR and DSR. The values of the No/No hybrid group were null for ISR and DSR like Takfa1 and S7328 genotypes as negative checks for those traits. This showed the importance of allele dosage of *R1-nj* accumulated during hybrid formation that *R1-nj*/*R1-nj* resulted in the highest expression of kernel *R1-nj* anthocyanin up to 100% followed by *R1-nj*/*r1-nj* with 40%–60% reductions and *r1-nj*/*r1-nj* with 100% reduction (**Figure 4**). The hybrid means of Tr/BHI group had better agronomic performance than inbred inducer BHI306 such as taller PH, similar AD, longer EL, and higher KW and TK, indicating that hybrids derived from this group may be useful for haploid induction in isolation fields.

**Table 5:** Means of inbred and hybrid inducers on representative agronomic traits, the *R1-nj* seed set, and haploid induction rate (HIR) evaluated in the dry season of 2021/2022.

Group	N^8^	PH (cm)	AD (DAP)	EL (cm)	KW (g)	TK	ISR (%)	DSR (%)^1^	HIR (%)^1^
p-Value		<0.001	<0.001	<0.001	<0.001	<0.001	<0.001	<0.001	<0.001
Tr/BHI^2^ hybrid inducers	10	187.5 b	52.7 e	15.4 a	105.2 d	321.0 d	98.9 a	87.8 a	2.9 b
Tr/Tr^3^ hybrid inducers	20	206.0 a	58.2 d	15.4 a	114.8 cd	381.2 c	90.7 c	75.4 b	1.8 c
No/BHI^4^ hybrids	4	193.8 b	52.7 e	14.9 a	102.4 d	343.6 cd	47.2 e	21.4 c	0.0 d
No/Tr^5^ hybrids	20	214.3 a	60.1 c	16.0 a	125.2 bc	422.0 b	50.9 d	24.7 c	0.0 d
No/No^6^ hybrids	2	217.9 a	60.5 c	16.0 a	150.9 a	485.7 a	0.0 f	0.0 d	0.0 d
Tropical inbred inducers	5	161.2 c	63.2 b	12.1 bc	58.1 f	241.8 e	94.2 b	82.4 a	1.7 c
BHI306^7^		125.1 d	51.3 e	11.0 c	23.9 g	143.3 f	100.0 a	73.4 b	4.3 a
Takfa1^7^		157.0 c	65.3 a	12.7 b	72.0 e	334.5 d	0.0 f	0.0 d	0.0 d
S7328^7^		215.2 a	64.7 a	16.4 a	136.9 b	468.4 a	0.0 f	0.0 d	0.0 d

Means with different letters in the same column are significantly different by Duncan’s multiple range test (DMRT) at 0.05 probability level.

PH, plant height; AD, anthesis date; EL, ear length; KW, total kernel weight per ear; TK, total kernel number per ear; ISR, the R1-nj inducer seed set; DSR, the R1-nj donor seed set.

^1^ Derived from donor genotype P789.

^2^ Tropical inbred inducers × BHI306 including the reciprocals.

^3^ Among tropical inbred inducers including the reciprocals.

^4^ Non-inducer genotypes × BHI306 including the reciprocals.

^5^ Non-inducer genotypes × tropical inbred inducers including the reciprocals.

^6^ Among two non-inducer genotypes including the reciprocals.

^7^ Standard checks.

^8^ The number of genotypes within group.

## Discussion

### Seasonal variation and the necessity of a multi-season trial in tropical savanna regions

In the TS of Thailand, the rainy season is characterized by higher temperature, precipitation rate, relative humidity, and longer photoperiod, while the dry season is identified by lower temperature and relative humidity, a lack of rainfall, and shorter photoperiod (Janket et al., 2018; Sintanaparadee et al., 2022). In our study, the significant effects of season and the interaction between season and genotype (G × S) for most traits observed suggest seasonal variation in TS climate and a crossover performance of haploid inducer genotypes between the rainy and dry seasons for agronomic traits and haploid induction ability. Sintanaparadee et al. (2022) examined the effects of seasonal variation in TS of Thailand and suggested that haploid induction in the dry season would be more efficient than that in the rainy season because Stock 6-based haploid inducer lines showed better tropical adaptation including seed set, flowering behaviors, and HIR.

Both season × GCA and season × SCA interactions were significant, indicating that performing hybrid formation and testing in a single environment might mislead the genotype selection. In maize and vegetable corn, hybrid evaluation through combining ability studies in TS of Thailand should be carried out in at least two different growing seasons to improve the accuracy of selection (Dermail et al., 2020; Fuengtee et al., 2020; Dermail et al., 2022; Ja et al., 2022). For practical reasons, different strategies should be considered by breeders when producing haploids in the different growing seasons. In the rainy season of TS, when the inducer plants seem to underperform like a shorter plant, smaller tassel and pollen production, shorter pollen-shed duration, and lower seed set, it is suggested to perform excess haploid induction crosses to retain an adequate number of haploid seeds as previously targeted. Consequently, additional induction crosses come to more inducer and donor plants that should be grown. Maintaining the seed stocks of both hybrid inducers and the parents is relatively difficult during this season; thus, it can be performed in the dry season. In the dry season, both producing haploids and maintaining inducer stocks are feasible.

### Reciprocal cross effects and their minor contributions to traits important for haploid inducers

The importance of reciprocal crosses in maize hybrid formation is still arguable. Previous studies on maize yield reported that reciprocal cross effects were non-significant, unstable across different genotypes, and negligible (Crane and Nyquist, 1967; Jumbo and Carena, 2008; Machida et al., 2010). In contrast, other studies noticed the significant contributions of reciprocal crosses in conditioning maize hybrid yield and proposed breeders to include the reciprocals during hybrid formation (Dimmock and Donovan, 1956; Derera et al., 2008; Mukanga et al., 2010; Dermail et al., 2018). In our study, reciprocal cross effects were significant but only marginally contributed to the total phenotypic variance for all traits observed except for ear diameter. Those opposite findings may be explained by different genetic backgrounds, mating designs, and the methods used to quantify those effects.

Reciprocal cross effects are attributable to maternal and non-maternal effects. The former is due to cytoplasmic genetic factors, while the latter is caused by the interaction between nuclear genes and cytoplasmic gene effects (Evans and Kermicle, 2001). Our finding revealed that the relative importance of the non-maternal effect was higher than the maternal effect for most observed traits including HIR. Lashermes and Beckert (1988) noticed that HIR showed no cytoplasmic effect but that it is nuclear in origin with the possibility of nuclear–cytoplasmic interactions. Recent studies confirmed that HIR is a male gametophytic trait (Kelliher et al., 2017; Zhong et al., 2019). The predominance of non-maternal over maternal effects was also reported on maize agronomic, yield, and quality traits (Kalsy and Sharma, 1972; Machida et al., 2010). The limited amount of DNA in chloroplasts and mitochondria and many genotype combinations might explain small maternal effects on most observed traits. However, the exception on EL, ED, and ISR might be due to the cytoplasmic and physiological effects of the female parent (Garwood et al., 1970).

Breeding haploid inducers *via* the traditional method is resource-intensive since each inducer genotype must be crossed to the donor females to check the percent HIR and self-pollinated to evaluate the per se performance. Since reciprocal cross effects are less important and reciprocal crosses may not be carried out, the number of hybrid crosses and further haploid induction to evaluate the HIR will significantly be reduced. In addition to saving workload and resources, breeders have the flexibility to assign pairwise parents for forming hybrid inducers since both cross directions (normal vs. reciprocals) will generate similar hybrids. Thus, the only concern is that the ideotypes and good nicking of pairwise parents appeal to hybrid seed production.

### Trait-dependent gene action and the breeding strategy on traits important for haploid inducers

HIR and the *R1-nj* expression are two major traits in breeding haploid inducers. However, no combining ability studies reported gene action controlling those traits. HIR is a heritable trait (Lashermes and Beckert, 1988; Almeida et al., 2020) and shows additive inheritance (Prigge et al., 2012b). Most studies in maize involving different genetic backgrounds, mating designs, and testing environments reported that additive genetic effects exceeded non-additive effects for grain yield, anthesis date, silking date, plant height, and ear height (Badu-Apraku et al., 2015; Abera et al., 2016; Fan et al., 2016; Adebayo et al., 2017; Zhang et al., 2017; Meseka et al., 2018; Yong et al., 2019; Maphumulo et al., 2021). In addition, additive effects were also important for ear diameter, row number per ear, and 1,000-kernel weight, while both additive and non-additive effects were equally important for ear length, kernel number per row, kernel number per ear, and kernel weight per plant (Yu et al., 2020). In contrast, Bhatnagar et al. (2004) and Machida et al. (2010) found the predominance of non-additive effects controlling maize grain yield.

Baker’s ratio represents the relative importance of GCA and SCA to predict hybrid performance. The ratio ranges from 0 to 1 where the closer the value is to 1, the higher the reliability of hybrid prediction based on the GCA alone (Baker, 1978). This ratio is useful when breeders found both significant GCA and SCA in their analysis, but they are still eager to know whether GCA or SCA is a more important controlling trait of interest. If a trait has Baker’s ratio closer to 1, it means that hybrid means can be predicted based on both parental line per se and the GCA and that trait is largely controlled by additive effect. It also can indicate the time of selection in a breeding program. Breeders may perform selection at early generations for traits under additive effects with Baker’s ratio close to 1, while later selection can be performed for traits under non-additive effects with Baker’s ratio close to 0. In our study, additive gene action with moderate-to-high heritability estimates was noticed for HIR, the *R1-nj* expression in both inducer and donor kernels, ear position, and flowering time (**Table 3**). It was implied that those traits were heritable, and the favorable alleles can be fixed at the early generation of an inducer breeding cycle (F_2_–F_3_). Then, since the remaining traits including plant height and yield components had poor-to-low heritability estimates and were controlled by both additive and dominance, phenotypic selection on those traits could be performed at later stages (F_4_ onward). Uliana Trentin et al. (2020) proposed a flexible tandem selection to fix *R1-nj*, *Pl1*, *mtl*, and *zmdmp* in F_2_–F_3_ generations; to discard F_3_ families with poor HIR through culling; and to improve agronomic performance in F_4_ or later generations through index selection. Chaikam et al. (2018) suggested that marker-assisted selection for *qhir1* can be performed on F_2_ or BC_1_F_1_ plants and that phenotypic selection for the *R1-nj* marker expression and agronomic traits can be carried out in F_5_ or BC_1_F_4_ generations.

### The relation between GCA and line per se and their possibility to predict the hybrid performance on traits important for haploid inducers

Plant breeding is a numbers game (Araus and Cairns, 2004), meaning that the number of hybrids tested increases exponentially with the increase of parental lines. It becomes time-consuming and resource-intensive. The possibility to predict hybrid performance based on the performance of parental lines represented by line per se and GCA has been explored. Simple linear correlation and linear regression are two common methods to determine the prediction accuracy of GCA estimates based on line per se and hybrid performance based on mid-parent (MP) values (Makumbi et al., 2011). To date, no studies reported inbred–GCA and inbred–hybrid relationships for HIR and other properties related to haploid inducers. For maize grain yield, Welcker et al. (2005) found a significant and close correlation between line per se and GCA estimates. In contrast, Ertiro et al. (2013) did not notice any significant inbred–GCA relation for grain and stover yield. Hybrid prediction based on MP values for maize grain yield was either significant (Argillier et al., 2000; Makumbi et al., 2011; Li et al., 2021) or not (Ertiro et al., 2013) in past studies. Accuracy might be determined by several factors: 1) gene action controlling the traits of interest, 2) the nature of the traits, 3) genetic distance among parental lines, 4) the level of inbreeding, and 5) growing environments. Additive effect on a particular trait led to high accuracy (Betrán et al., 2003), while non-additive including dominance effects contributed to poor prediction (Hallauer et al., 2010). The prediction accuracy varied across traits from poor for complex traits such as maize grain yield to high for less complex traits such as plant height and silking date (Gowda et al., 2013; Adebayo et al., 2017). The wider genetic distance among lines resulted in more efficient MP-based hybrid prediction (Charcosset et al., 1998). Under stress environments such as drought and acid soils, the inbred–hybrid correlations for maize grain yield were lower than those under optimum conditions (Betrán et al., 2003; Welcker et al., 2005). The level of inbreeding also contributed to the poor correlation since inbred lines were more susceptible to stress and performed worse than early breeding lines (Betrán et al., 2003).

In our study, the inbred–GCA correlation coefficients were significant and high (0.73*< r< 0.99**) on most observed traits including HIR. Although the inbred–hybrid linear regression was significant for all observed traits except ear diameter, the coefficients varied across traits: high for flowering time and the *R1-nj* seed set of inducers; moderate for plant architecture, ear weight, total kernel weight per ear, total kernel number per ear, and HIR; and low for ear length. The significance of the inbred–GCA correlation followed by the inbred–hybrid relation was due to additive gene action predominantly controlling traits observed. Hybrid performance is derived from the sum of the MP heterosis and the MP value (Li et al., 2021) and is controlled by the additive, the dominance, and the epistatic effects (Jiang et al., 2017). According to our findings, we propose to use line per se data to predict GCA estimates and to use MP values to predict hybrid performance for flowering time and *R1-nj* seed set of inducers only. However, the MP values could not substitute hybrid evaluation through field trials for other traits including HIR. The accuracy for HIR was limited because HIR is a male gametophytic trait (Kelliher et al., 2017; Zhong et al., 2019) and sensitive to reduction by pollen contamination, which has selection advantages over pollen of inducers (Chaikam et al., 2019). Therefore, to enhance the hybrid prediction based on MP values, both male and female lines should have equal alleles responsible for HIR such as *mtl* and *zmdmp* prior to estimating the MP. It can be performed by selecting pairwise parents both performing high per se and good GCA for HIR.

### The significance of heterosis for breeding hybrid inducers

Heterosis reflects the better performance of F_1_ progenies compared to their corresponding parents. It has been exploited for developing maize hybrids with superior traits such as high yield, early maturity, drought tolerance (Adebayo et al., 2017), and pest and disease resistance (Kasoma et al., 2021; Maphumulo et al., 2021). In our study, the magnitudes of heterosis, both MPH and BPH, varied across observed traits. Traits controlled by both additive and dominance effects such as ear weight, total kernel weight per ear, and total kernel number per ear showed higher estimates of heterosis, while traits controlled by additive effects such as flowering time and ear height had relatively low heterosis. Gene interactions influencing heterosis and hybrid mean for certain traits were different. Regarding grain yield and yield components, Li et al. (2021) found that high heterosis was due to dominance while the high hybrid mean was due to epistasis.

The genetic distance of the parental pair also contributes to the level of heterosis attained. The wider genetic divergence resulted in higher heterosis to some extent before the value decreased in crosses between extremely divergent lines (Moll et al., 1965). Stupar et al. (2008) argued that genetic distance can only predict poor hybrids but not superior hybrids because the reliability to predict heterosis decreased when performing hybrid formation derived from distantly related parents. It is assumed that the high variation of heterosis especially for yield-related traits in our study was caused by various degrees of relatedness since a full-diallel mating scheme allowing crosses both intra-ecotype (tropical × tropical) and inter-ecotype (tropical × temperate) was performed. Yu et al. (2020) reported that temperate × tropical crosses showed higher levels of heterosis than intra-ecotype (temperate × temperate or tropical × tropical) crosses on several yield-related traits.

In our study, the level of heterosis of each trait was higher in the rainy season than in the dry season. In the tropical savanna of Thailand, the rainy season was unfavorable for growing maize haploid inducers due to high temperature, precipitation, and relative humidity. When the weather during the rainy season is getting worse, the parental lines become more susceptible to biotic and abiotic stresses than the hybrids due to the nature of inbreeding depression. Makumbi et al. (2011) noticed higher heterosis for grain yield in drought conditions than in the well-watered condition.

Typical maize hybrids in tropical regions are high-yielding are early maturing and have short plant stature (Abadassi, 2015; Dermail et al., 2022). Thus, positive heterosis for grain yield and yield components and negative heterosis for flowering time and plant architecture are favorable. However, the maize ideotype of hybrid inducers in maternal haploid production under isolation fields should be taller than the female donor to optimize the pollination and seed set. In that case, high hybrid means and positive heterosis for plant height are preferred. Our study showed that hybrid inducers derived from either tropical/temperate or tropical/tropical inducer lines had taller plant stature than the corresponding inbred inducers. The predominance of negative heterosis for HIR was contributed by the unequal presence of alleles responsible for HIR. In this study, 26 of 56 crosses involving non-inducer lines, either Takfa1 or S7328 as parents, exhibited no HIR, comparable with the negative checks for HIR. Although there were no hybrid inducers that could surpass the HIR of standard check BHI306, the F_2_ populations derived from tropical × temperate inducer crosses may be further improved and selected for higher HIR. Introgression of favorable alleles responsible for HIR from temperate maize BHI306 into the tropical background can hopefully broaden the genetic variation of tropical maize on haploid induction ability.

First, to optimize haploid production *via in vivo* maternal system under isolation fields, hybrid inducers should be able to induce haploids if both parents have equal recessive alleles for HIR. Second, to facilitate haploid identification at the kernel stage, both parents should also possess dominant alleles for *R1-nj*. Third, the plants of hybrid inducers may exhibit high heterosis for agronomic traits, which is proven in this study. Hybrid inducers are expected to be more vigorous and to produce more pollen than inbred inducers, making the haploid induction process more convenient. If so, higher haploid yields as well as resource-saving per induction season can be achieved.

## Conclusions

Inducer selection for HIR, the *R1-nj* marker expression, ear position, and flowering time can be performed at the early stage. Once inducers are fixed for those traits, further selections for plant height and yield components may be performed at later stages (F_4_ onward). Temperate inducers had the genetic potential for improving HIR and the *R1-nj* marker expression, while tropical non-inducers contributed to sharing favorable alleles for good agronomic performance. The concept of haploid induction in isolation fields will be feasible and convenient if hybrid inducers can express maximum heterosis for agronomic traits while maintaining the ability to produce haploids and facilitating haploid selection *via* the *R1-nj* marker. Finally, reducing the number of crosses regarding lacking reciprocal effects and pre-selected mid-parents on both sides fixed for the *R1-nj* and HIR alleles will enhance the efficiency of breeding hybrid inducers.

## Data availability statement

The data that support the findings of this study are available from the corresponding author upon reasonable request.

## Author contributions

KL, KS, and AD: conceptualization. KS, SC, and AD: methodology. AD and WS: data analysis and interpretation of the results. AD: writing original draft preparation. TL, KS, WS, VR, and SC: supervision, review, and editing. KS and VR: funding acquisition. All authors contributed to the article and approved the submitted version.
